# Expanding the expanded risk score to psoriatic arthritis: any port in a storm

**DOI:** 10.1093/rheumatology/keaf209

**Published:** 2025-04-21

**Authors:** Ennio Giulio Favalli, Luca Ingrao, Giorgia Trignani, Matteo Ferrito, Gilberto Cincinelli, Roberto Caporali

**Affiliations:** Department of Rheumatology and Medical Sciences, ASST Gaetano Pini-CTO, Milan, Italy; Department of Clinical Sciences and Community Health, Università degli Studi di Milano, Milan, Italy; Department of Rheumatology and Medical Sciences, ASST Gaetano Pini-CTO, Milan, Italy; Department of Clinical Sciences and Community Health, Università degli Studi di Milano, Milan, Italy; Department of Rheumatology and Medical Sciences, ASST Gaetano Pini-CTO, Milan, Italy; Department of Clinical Sciences and Community Health, Università degli Studi di Milano, Milan, Italy; Department of Rheumatology and Medical Sciences, ASST Gaetano Pini-CTO, Milan, Italy; Department of Clinical Sciences and Community Health, Università degli Studi di Milano, Milan, Italy; Department of Rheumatology and Medical Sciences, ASST Gaetano Pini-CTO, Milan, Italy; Department of Clinical Sciences and Community Health, Università degli Studi di Milano, Milan, Italy; Department of Rheumatology and Medical Sciences, ASST Gaetano Pini-CTO, Milan, Italy; Department of Clinical Sciences and Community Health, Università degli Studi di Milano, Milan, Italy

Rheumatology key message

- The use of a PsA-specific CV score can be a useful tool in clinical practice.

Dear Editor, Patients with psoriatic arthritis (PsA) exhibit a heightened risk of developing cardiovascular disease (CVD) compared with the general population. This increased risk results from a combination of a higher prevalence of traditional cardiovascular (CV) risk factors (such as hypertension, obesity, metabolic syndrome and dyslipidaemia), chronic inflammation and the use of drugs commonly associated with increased CV risk, such as corticosteroids and NSAIDs [1]. Therefore, employing an appropriate CV risk definition score is imperative in PsA patients to both prevent CV complications and to comply with recent European Medicines Agency (EMA) guidance on restriction of Janus Kinase inhibitors (JAKis) prescription in patients carrying CV risk factors [2]. Nevertheless, to date a specific CV algorithm for PsA has not yet been developed and EULAR still recommends using existing general population prediction models multiplied by a correction factor of 1.5 for patients with PsA [3]. A recent study compared the performance of four scoring systems in patients with PsA, the Systematic Coronary Risk Assessment (SCORE), the modified version proposed by EULAR (mSCORE), an updated version of SCORE (SCORE2) and the QRESEARCH risk estimator version 3 (QRISK3) [4]. QRISK3 and SCORE2 yielded promising results in identifying PsA patients at high risk for CV events, but none of these adequately addressed disease-specific factors that contribute to further CV risk increase, such as disease activity and duration and/or corticosteroid use. Furthermore, the calculation of all the above scores requires precise cholesterol plasma levels, which are often unavailable during a routine rheumatology examination.

We have recently evaluated the Expanded Cardiovascular Risk Score in rheumatoid arthritis (ERS-RA) [5] as a feasible and accurate tool to assess CV risk in a cohort of rheumatoid arthritis (RA) patients treated with JAKis [6]. To explore the applicability of ERS-RA to PsA as a potential disease-specific CV score (ERS-PsA) for assessing eligibility for JAKis, we conducted a cross-sectional study in an Italian monocentric cohort of adult PsA patients recruited from September 2022 to June 2023 and treated with targeted therapies. The project was approved by the Ethics Committee of the Geatano PIni Institute with approval number 141/2010. All included patients have signed an informed consent to participate in the data collection.

In our proposal of ERS-PsA, we replaced the Simplified Disease Activity Index (SDAI) used in RA with the Disease Activity in Psoriatic Arthritis (DAPSA) score specifically for PsA as an indicator of disease activity. The choice of the DAPSA among the available disease activity measurement tools was determined by the simplicity of calculation, which makes it the most commonly used in clinical practice as opposed to potentially more comprehensive but difficult-to-use scores such as the Psoriatic Arthritis Disease Activity Score (PASDAS). Patients were considered ineligible for JAKis if they met the EMA criteria, which include being over 65 years, being a current or former smoker, or carrying an increased risk of major adverse cardiovascular events (MACE) or malignancy. The MACE risk was defined either according to ORAL Surveillance trial [7] inclusion criteria (ORALSURV) or as >10% risk of CV events in 10 years calculated by ERS-PsA.

Out of 369 PsA patients (baseline characteristics are reported in Table 1), 163 (44.1%) were identified as having an increased CV risk based on the ORALSURV inclusion criteria and only 98 (26.5%) based on the ERS-PsA criteria (*P* < 0.001). Using either of these CV risk definitions, 229 (62%) versus 174 (47.1%) patients were found to be ineligible for JAKis according to EMA/ORALSURV or EMA/ERS-PsA criteria, respectively (*P* < 0.001). At the time of our analysis, 9 of 369 patients were on JAKis therapy and, of these, 5 would have been ineligible according to the EMA-ORALSURV criteria and 4 according to the EMA-ERS-PsA criteria.

**Table 1.** Characteristics of the population

Variables	Overall population (*N* = 396)	EMA-ORALSURV (*N* = 229)	EMA-ERS-PsA (*N* = 174)
Years (mean ± SD)	53.3 ± 11.9	57.5 ± 11.5	57.9 ± 12.9
Male sex, *n* (%)	191 (51.8%)	125 (54.6%)	94 (54.0%)
BMI (mean ± SD)	25.5 ± 4.3	25.8 ± 4.5	25.5 ± 4.6
Overweight (BMI > 25), *n* (%)	76 (48.4%)	116 (50.7%)	82 (47.1%)
Obese (BMI >30), *n* (%)	47 (12.9%)	35 (15.3%)	23 (13.2%)
Smokers, *n* (%)	97 (26.2%)	97 (42.4%)	97 (55.7%)
Arterial hypertension, *n* (%)	101 (27.4%)	70 (30.6%)	62 (35.6%)
CAD, *n* (%)	12 (3.3%)	12 (5.2%)	10 (5.7%)
Dyslipidaemia, *n* (%)	113 (30.6%)	104 (45.4%)	68 (39.1%)
Diabetes mellitus, *n* (%)	26 (7.0%)	26 (11.4%)	24 (13.8%)
Malignancy, *n* (%)	20 (5.4%)	20 (8.7%)	20 (11.5%)
Disease duration, years (mean ± S.D.)	14.4 ± 9.5	13.89 ± 10.3	14.4 ± 10.3
Corticosteroid users, *n* (%)	49 (13.3%)	27 (11.8%)	28 (16.1%)
NSAIDs users, *n* (%)	158 (42.8%)	95 (41.5%)	73 (42.0%)
JAKi, *n* (%)	9 (2.4%)		
JAKi as first-line targeted therapy, *n* (%)	1 (0.3%)		
JAKi in pts refractory to ≥2 MoA, *n* (%)	5 (1.4%)		
bDMARDs as first-line therapy, *n* (%)	217 (58.8%)		
bDMARDs in resistant to ≥2 MoA, *n* (%)	69 (18.7%)		

bDMARD: biologic disease modifying anti-rheumatic drug; CAD: coronary artery disease; JAKi: Janus-Kinase Inhibitor; MoA: mechanism of action.

In conclusion, this study presents for the first time a new version of the ERS-RA score adapted for calculating CV risk in PsA. As we have already reported for RA [6], the use of a disease-specific score such as the ERS-PsA allows for an accurate calculation of CV risk in patients eligible for JAKis, minimizing the proportion of ineligible subjects compared with the ORALSURV criteria [7]. Moreover, given the high prevalence of CV events in PsA, ERS-PsA could be proposed as a tool for better determination and monitoring of CV risk, also in patients on therapy with alternative mechanisms of action to JAK blockade. Exactly as happened with the ERS-RA in the US registry CORRONA [5], we hope that the ERS-PsA will also undergo a validation process on a larger cohort more representative of the general population of PsA patients, so that its large-scale use in clinical practice can then be considered.

## Data Availability

Study protocol, statistical analysis and individual participant data that underlie the results reported in this article, after deidentification (text, tables, figures and appendices), will be available beginning 6 months and ending 5 years following article publication.

## References

[1] Scriffignano S , Lories R, Toukap AN et al Cardiovascular comorbidities in psoriatic arthritis: epidemiology and risk factors in two different European populations. Clin Exp Rheumatol 2023;41:1815–22. 36826796 10.55563/clinexprheumatol/aovika

[2] EMA. Janus kinase inhibitors (JAKi). Amsterdam: European Medicines Agency, 2022. https://www.ema.europa.eu/en/medicines/human/referrals/janus-kinase- inhibitors-jaki.

[3] Agca R , Heslinga SC, Rollefstad S et al EULAR recommendations for cardiovascular disease risk management in patients with rheumatoid arthritis and other forms of inflammatory joint disorders: 2015/2016 update. Ann Rheum Dis 2017;76:17–28. 27697765 10.1136/annrheumdis-2016-209775

[4] Polo Y La Borda J , Castañeda S, Heras-Recuero E et al; CARMA Project Collaborative Group. Use of risk chart algorithms for the identification of psoriatic arthritis patients at high risk for cardiovascular disease: findings derived from the project CARMA cohort after a 7.5-year follow-up period. RMD Open 2024;10:e004207. 38631846 10.1136/rmdopen-2024-004207 PMC11029293

[5] Solomon DH , Reed GW, Kremer JM et al Disease activity in rheumatoid arthritis and the risk of cardiovascular events. Arthritis Rheumatol 2015;67:1449–55. 25776112 10.1002/art.39098 PMC4446181

[6] Favalli EG , Cincinelli G, Germinario S et al The impact of EMA recommendations on the real-life use of Janus kinases inhibitors for rheumatoid arthritis: the Expanded Risk Score in RA as a tool to quantify the risk of cardiovascular events. Front Immunol 2023;14:1225160. 37720218 10.3389/fimmu.2023.1225160 PMC10500057

[7] Ytterberg SR , Bhatt DL, Mikuls TR et al; ORAL Surveillance Investigators. Cardiovascular and cancer risk with tofacitinib in rheumatoid arthritis. N Engl J Med 2022;386:316–26. 35081280 10.1056/NEJMoa2109927

